# Retraction Note: Inequities in coverage of smokefree space policies within the United States

**DOI:** 10.1186/s12889-018-5716-y

**Published:** 2018-06-26

**Authors:** Christopher Lowrie, Amber L. Pearson, George Thomson

**Affiliations:** 1 Department of Geography, Environment, and Spatial Sciences, Michigan State University, East Lansing, MI 48824 USA; 2 Department of Public Health, University of Otago, Wellington, 6021 New Zealand; 3 Environmental Science and Policy Program, Michigan State University, East Lansing, MI 48824 USA

## Retraction

The authors have retracted this article [1] because of an error with the data extraction process. A re-examination of the dataset suggests that the authors did not include all smokefree policies for indoor spaces. Due to the error with the data extraction process, the conclusions drawn in the article regarding smokefree policies for indoor spaces are incorrect. All authors agree to this retraction. A revised version of the article has now been published [2].
